# The effect of non-optimal ambient temperature on daily mortality in Colombia 2010–2019

**DOI:** 10.1007/s11869-025-01782-9

**Published:** 2025-06-27

**Authors:** Nicolas Borchers-Arriagada, Antonio Gasparrini, Laura A. Rodriguez-Villamizar

**Affiliations:** 1 Menzies Institute for Medical Research, University of Tasmania, Tasmania, Australia; 2 Environment & Health Modelling Lab, Department of Public Health, Environments and Society, London School of Hygiene & Tropical Medicine, London, UK; 3 Departamento de Salud Pública, Escuela de Medicina, Universidad Industrial de Santander, Carrera 32 29-31 Of. 301, 680002 Bucaramanga, Colombia

**Keywords:** Temperature, Extreme heat, Time series, Mortality, Attributable risk, Colombia

## Abstract

Adverse effects of non-optimal temperatures on mortality have been reported in different climates. However, only a low number of studies have been conducted in tropical locations where these effects might differ. Here, we estimate the association between ambient air temperature and all-cause mortality and assess the impact of non-optimal temperatures on attributable mortality at national and subnational levels in Colombia during 2010–2019. We obtained daily temperature and mortality data for 32 departments and conducted a two-stage analysis. In stage-1 we fitted a time-series Poisson model for each department and estimated the mortality-temperature association using distributed-lag-nonlinear models with 7–28 days of lag. In stage-2, we pooled these estimates using a multivariate meta-analytic model including mean temperature, relative humidity, and a multidimensional poverty index. We calculated attributable deaths and fractions due to non-optimal temperatures and due to overall heat and cold. We analyzed 2,561,561 deaths and found marked differences in exposure–response curves of mortality-temperature, where most departments showed acute heat effects but no cold effects for the 7-days lag. This lag-response curve for heat showed that the risk of death is higher during the same day (lag 0) of extreme temperatures and decreases after the third day. The country attributable fraction due to non-optimal temperature was higher for heat (1.77, 95% CI 1.16 – 2.31) during the 0–7 days lag, but higher for cold temperatures (4.68, 95% CI 2.34 – 6.72) during the 0–28 days lag. There was high heterogeneity in the estimated risks between departments. These results should inform planning adaptation strategies for climate change differentiated at subnational level.

## Introduction

Climate change is the priority environmental challenge worldwide. The rise in sea-level, increasing temperatures, flooding, and wildfires, among others, are impacting human health (IPCC 2023). The main health impacts include increasing incidence of infectious and cardio-respiratory diseases, undernutrition, and heat-related morbidity and mortality (Rocque et al. 2021; Watts et al. 2021).

The health-related effects of climate change are present in all continents and are having a disproportionate impact on populations. A recent multi-country study estimated that on average, 37% of warm-season heat related deaths can be attributed to human-induced warming during 1991–2018, which translates into more than 9,700 deaths from 732 locations in 43 countries (Vicedo-Cabrera et al. 2021). Moreover, the health-related effects of climate change are having an unequal impact on populations around the world. A higher proportion of heat-related mortality attributed to human-induced climate change was found in South American countries, particularly in Ecuador, Colombia, and Perú (Vicedo-Cabrera et al. 2021). Countries in South America face heightened exposure and vulnerability to the impacts of climate change, compounded by preexisting challenges such as social inequalities, economic hardships, deforestation, and land degradation, all of which amplify the adverse health consequences of climate change (Hartinger et al. 2023). Also, a large proportion of global deaths have been associated not only with heat but with exposure to both cold and hot non-optimal temperatures (i.e., temperatures above or below the minimum mortality level) (Burkart et al. 2021; Zhao et al. 2021). Zhao et al. (2021) estimated that between 2000–2019, 8.52% (95% CI, 6.19%—10.47%) of global deaths were associated with cold temperatures, while 0.91% (95% CI, 0.56% – 1.36%) with hot temperatures.

Colombia, a leading country in the Latin America and the Caribbean (LAC) region, is located in the equatorial zone where the impacts of heat might be more severe (Vicedo-Cabrera et al. 2021). In 2020, Colombia updated its Nationally Determined Contribution (NDC) aiming to reduce greenhouse gas (GHG) emissions by 51% by 2030, and to adapt to the impacts of climate change. In addition, the Colombia NDC identified needs for achieving the goals that included national-local research and analysis of information tools (República de Colombia 2020). A recent study assessed the benefits of rising goals in the Colombian NDC and found that implementing a high ambition mitigation scenario will not only reduce GHG emissions but will also prevent 3,800 deaths annually from reducing ambient air pollution by 2030 (World Health Organization 2023). As of today, however, there is no estimation of the health-related impact of non-optimal temperatures in terms of mortality at a subnational level to inform local decisions and policies in Colombia. Therefore, the objective of this study was to estimate the association between ambient air temperature and all-cause mortality and to assess the impact of non-optimal temperature on attributable mortality at a national and subnational level in Colombia during 2010–2019.

## Materials and methods

### Study area

**Fig. 1:**
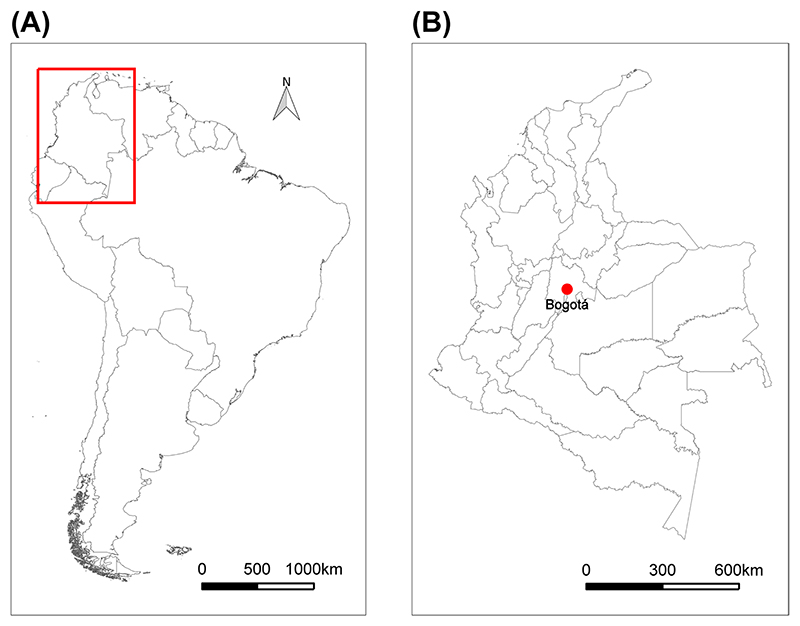
Map of: (**A**) Colombia within South America, (**B**) Departments within Colombia and capital city (Bogotá)

Colombia is a country located at the northwestern part of South America with an estimated population of 50 million inhabitants in 2019 (Departamento Nacional de Estadística (DANE) 2020). It is divided into 33 geographical units including 31 continental departments- main administrative geographical unit –, the capital district of Bogotá (Fig. 1) and one island department (San Andrés and Providencia). For this study, we only considered the 31 continental departments and the capital district of Bogotá for a total of 32 geographical units of analysis, hereafter referred to as departments.

### Data sources

We obtained daily counts of all-cause deaths between 1 January 2010 and 31 December 2019 by department of residence from the national vital statistics for social protection information system (SISPRO for its initials in Spanish, www.sispro.gov.co).

We obtained hourly temperature and dewpoint temperature at 2 m above the surface of the land at a 0.1° spatial resolution from the fifth generation ECMWF reanalysis (ERA5) hourly data on single levels (Hersbach et al. 2023). For each department, we calculated daily mean temperature and daily mean relative humidity at UTC-5 time zone. Relative humidity was calculated using the August-Roche-Magnus approximation.

We obtained 3-hourly fine particulate matter (PM_2.5_) concentration data from the Copernicus Atmospheric Monitoring System (CAMS) global reanalysis (EC4) dataset (Inness et al. 2019) at a 0.75° spatial resolution. We calculated daily mean PM_2.5_ concentrations for each department at UTC-5 time zone.

Population counts by department for each year were obtained from the population projection time series made available by the National Administrative Department of Statistics (DANE for its initials in Spanish, www.dane.gov.co).

We used the Colombian multidimensional poverty index (MPI) as a socioeconomic measure for our analysis as socioeconomic conditions might be related to mortality (Angulo Salazar et al. 2013). The MPI, developed by DANE in 2011, serves as a composite index that captures poverty from a multi-deprivation perspective. It encompasses five dimensions, including education level, conditions of children and youth, employment, health, access to public utilities, and housing conditions. For our study, we used the Colombian 2018 MPI, which falls within the study period and represents the most recent MPI measurement derived from national census data (Departamento Nacional de Estadística (DANE) 2022). The MPI indicates the percentage of the population experiencing multidimensional poverty. Hence, a higher index value corresponds to a greater degree of socioeconomic deprivation.

### Statistical analysis

We modelled the relationship between daily mean temperature and counts of all-cause mortality by using a two-stage analysis following Gasparrini et al. (2015). In the first stage we used a time-series regression with quasi-Poisson distribution and distributed non-linear model (DLNM) to estimate department-specific associations between temperature and mortality. In the second stage, we pooled the department-specific estimates derived from the first stage by using a multivariate meta-analysis. Later, we used the fitted meta-analytical model to estimate the best linear unbiased predictions (BLUP) for the department-specific overall cumulative exposure–response associations expressed as Relative Risks (RRs). Finally, we used these RRs to calculate the total number of attributable deaths and fraction of attributable deaths due to non-optimal temperature having as reference the department-specific optimum temperature (i.e., the temperature at which mortality is minimum). We conducted sensitivity analyses using different modelling choices, explained further below.

For the first stage we used a DLNM model using a natural cubic B-spline with 6 degrees of freedom (df) per year to control for seasonal and long-term trends, an indicator for day of the week, and a linear term to control for relative humidity. We selected this approach for its capacity to capture effects that are delayed in time and a non-linear relationship between exposure and response, as has been shown in previous studies (Armstrong 2006; Gasparrini et al. 2010). The sensitivity analyses included models with 4 and 8 df per year. The exposure response curve was modelled using a B-spline with three knots at the 10th, 75th, and 90th percentiles of the department-specific temperature distributions. We used a lag-period of 7 days in the main model to account for acute effects of non-optimal temperatures and did a sensitivity analysis with lag-periods of 14, 21, and 28 days to account for more extended periods of cumulative exposure. The selection of the 7-lag period accounting for short-term effects as our main model was based on the fact that this period is particularly relevant for informing early warning systems and immediate public health actions. The lag-response function was parameterized using natural cubic splines with two knots for the lag 0–7 analysis and three knots for the lag 0–14 analysis and beyond. We then calculated reduced department-specific estimates by calculating the overall cumulative risk during the lag-period for the temperature-mortality association. To account for potential changes in temperature-mortality associations over time, we ran a sensitivity analysis stratified by five-year periods: 2010–2014 and 2015–2019. Given there is still debate whether air pollution acts as a confounder and needs to be controlled for in temperature studies, we also did a sensitivity analysis controlling for air pollution, specifically PM_2.5_ (Buckley et al. 2014). A graphical representation of the methods is presented in Figure S1 in the supplementary material.

For the second stage, in the multivariable analytical model we included as meta-predictors the department-specific average temperature, temperature range (max - min), population weighted latitude and elevation, the average population density, average relative humidity, and the MPI to account for differences in socioeconomic conditions across departments. We used the Wald test to test the effect of the meta-predictors and the Cochrane *Q* test and *I*^*2*^ statistic to test the residual heterogeneity. The fitted meta-analytical model was used to estimate the BLUP of the overall cumulative exposure–response association for each department, as a more robust predictor that accounts for potential bias derived from small numbers in death counts by using information from the first-stage and second-stage estimations (Gasparrini et al. 2015). The BLUP was used to calculate the minimum mortality temperature (MMT) that was used as optimum temperature.

We calculated the attributable risk by re-centering the exposure–response model using the MMT as a reference and then we calculated the daily number of attributable deaths for each department. Then, we summed all daily counts of attributable deaths to calculate the total number of attributable deaths per department. The attributable fraction was calculated as the proportion of the total number of attributable deaths due to non-optimal temperature out of the total number of deaths. Specific attributable deaths and attributable fractions to cold and heat were calculated using the days with lower or higher values than the MMT. Finally, we estimated the attributable deaths and attributable fractions for the 1st, 5th, 10th, 90th, 95th, and 99th percentile distributions of temperature for each department to estimate the effect of colder and hotter mean temperatures. All analyses were done with the R software using the *dlnm* and *mixmeta* packages (Gasparrini 2011; R Core Team 2018; Sera et al. 2019).

## Results

Supplementary Table S1 shows descriptive statistics for mortality, temperature, relative humidity, PM_2.5_, MPI, latitude, elevation, and population data by department. There were 2,561,561 deaths across 32 departments during 2010–2019 included in our analysis, which corresponds to an average of about 256,000 deaths per year. There was great variation in mortality rates by department, with the highest annual mortality rates (> 7,000 deaths per 100,000 population) observed for Quindío, Huila, and Tolima. On the other end of the spectrum, Vichada, La Guajira, and Choco had the lowest annual mortality rates (< 3,000 deaths per 100,000 population). Temperature also varied substantially across departments with the lowest mean daily temperatures observed in the district of Bogotá (12.4ºC) in the central Andean region of the country, and the highest in Sucre (28.2ºC) in the north Atlantic region. Supplementary Figure S2 shows maps of the mean annual mortality rate and the mean annual temperature by department during the study period.

The exposure–response curves for each department, with their corresponding minimum mortality temperature and the cutoffs to define non-optimal temperatures (cold and heat), are presented in Supplementary Figures S3-S4. As expected, we found marked differences by department in the shape of the 7-day lag exposure–response curve of temperature and mortality. The risk of death increased with higher temperatures for almost all departments with the highest statistically significant RRs for Vaupés, Amazonas, Guainía, Guaviare, Vichada, Caquetá, Putumayo, Huila, Meta, and Quindío. Among these departments the minimum mortality temperature varied greatly, being the lowest in Quindío (15.5ºC) and the highest in Vichada (25.7ºC).

**Fig. 2:**
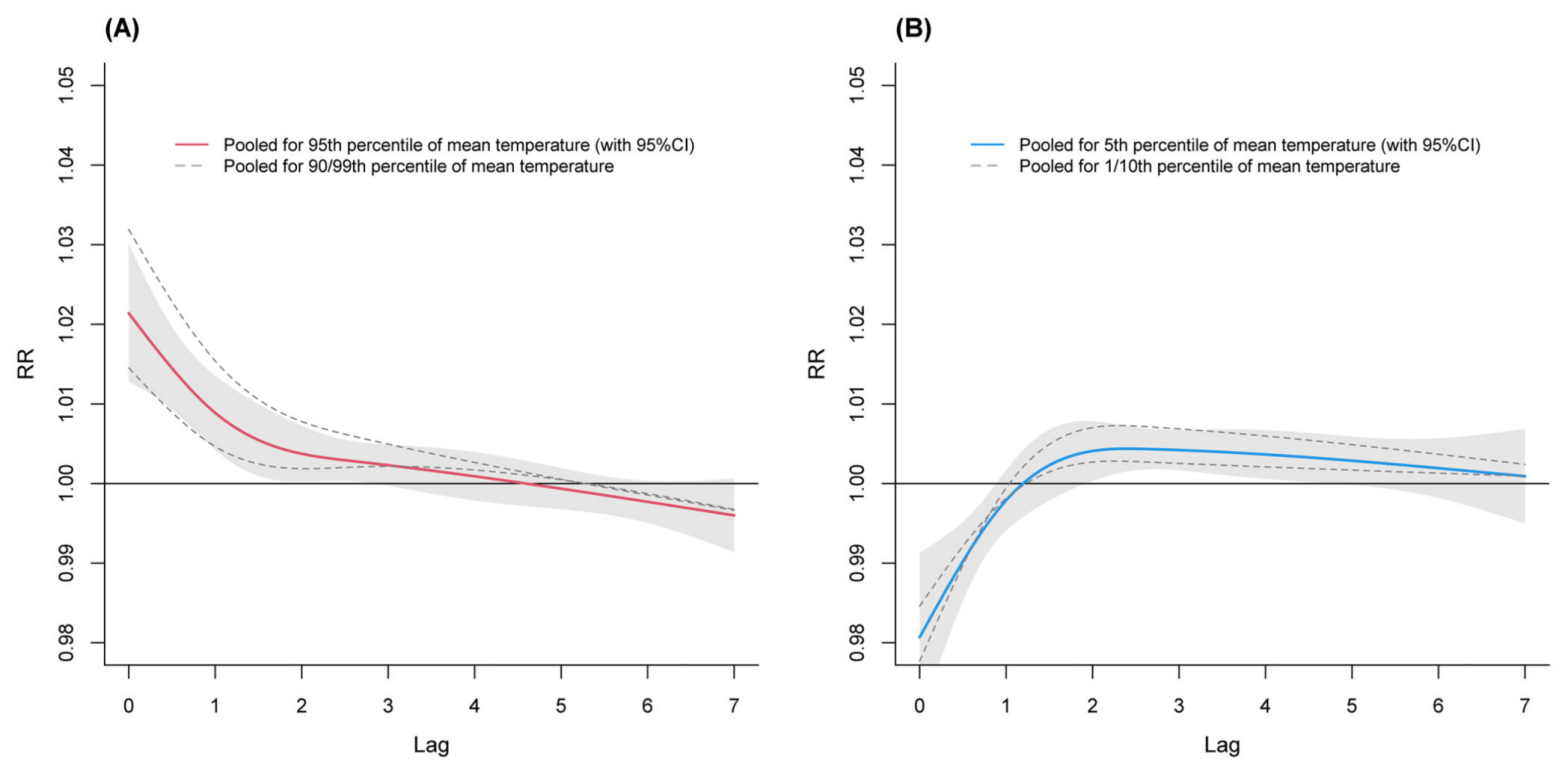
Pooled predictor-specific temperature-mortality association for Colombia using 7-day lag: **A**) Heat **B**) Cold

The pooled predictor-specific temperature-mortality association for Colombia for hot (A) and cold (B) temperatures for 7-days lag are presented in Fig. 2. The figure shows how the risk of death during hot temperatures is higher during the same day (lag 0) of maximum temperatures and decreases after the third day. For cold temperatures a different pattern is observed with the risk of death being negative at lag 0 and lag 1, peaking at around lag 4 and then decreasing again. The lag-response curves for 14-, 21-, and 28-days lag are presented in the supplementary material (Figures S5–S7).

The multivariate meta-regression results showed that residual heterogeneity was very low (I^2^ = 3.4%) and among all meta-predictors only population weighted elevation showed a statistically significant modified temperature-mortality association (Table S2).

**Fig. 3:**
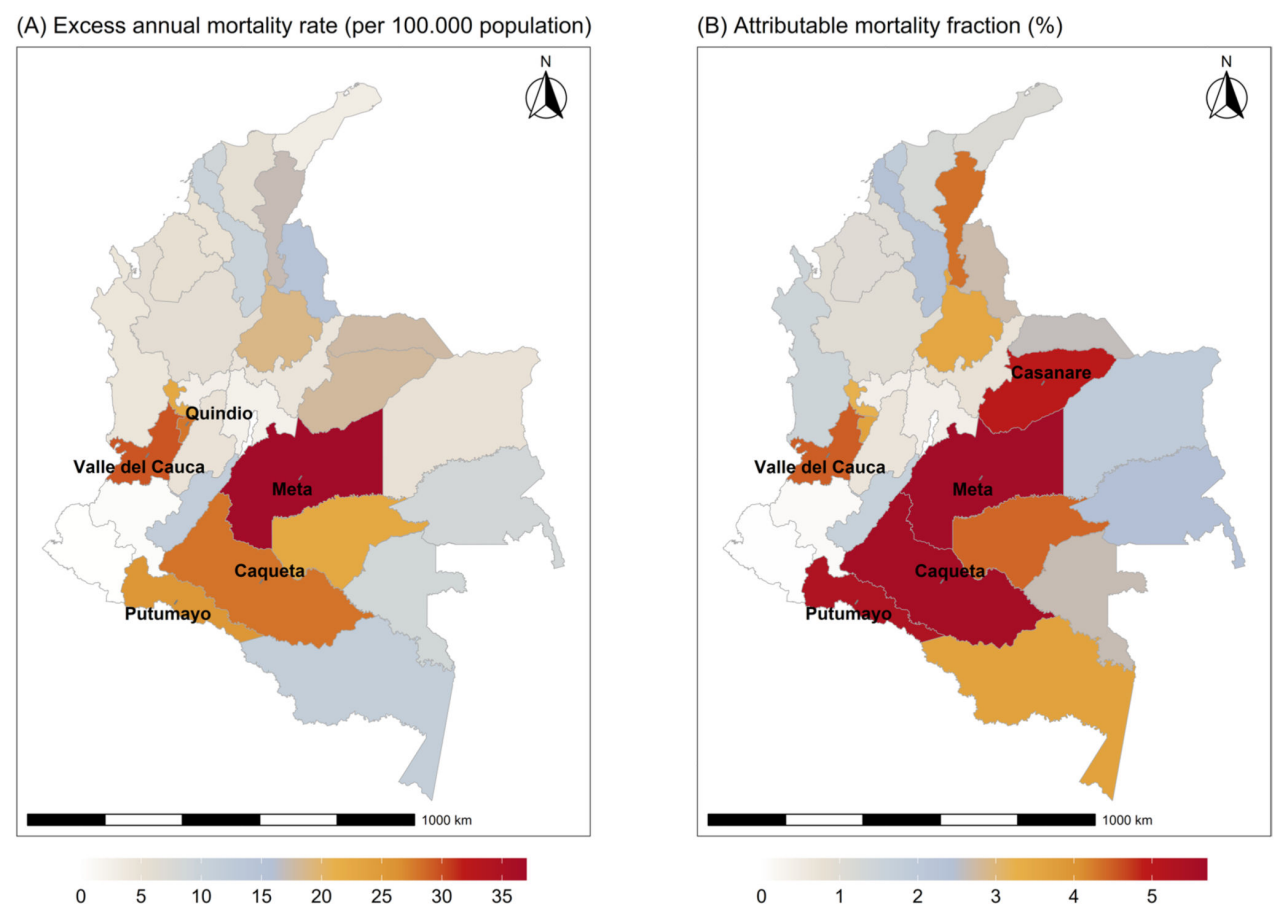
Map of excess annual mortality rate (per 100.000 population) (**A**) and attributable mortality fraction (%) (**B**) due to heat in Colombia, 2010–2019. NOTE: only the 5 departments with the highest mortality rate or attributable fraction are labelled

Total estimated attributable deaths and attributable mortality fractions are presented in Table 1. Overall, the attributable fraction due to non-optimal temperature was 2.45% (95% CI 1.42 to 3.30) of total mortality and the attributable fraction for heat was more than two times the fraction for cold temperatures during the 0–7 lag. Maps of excess mortality rate (per 100.000 population) and attributable mortality fraction due to non-optimal temperatures by departments are presented in Fig. 3 and results detailed in Table 2. The highest attributable mortality fraction was found in Meta (5.66%) and Caquetá (5.56%) while the lowest was found in Cundinamarca (0.78%) and Boyacá (1.03%). The minimum mortality temperatures ranged between 14.3 ºC in Bogotá and 27.8 ºC in Sucre, located at the center and north of the country, respectively. The median minimum mortality percentile for the country was 15 with a range between 1 and 99 among departments (Table 2).

The sensitivity analysis showed that when using different degrees of freedom choices in the DLNM we obtained similar results for the estimation of attributable fractions for the same lag period. In contrast, the selection of different lag periods changed the magnitude of attributable fractions due to heat and cold with higher heat effects compared to cold effects for acute lag periods (7- days) and higher cold effects for extended lag periods (14-, 21- and 28-days) (Table S3). Moreover, while the effect of heat on attributable mortality fraction remains relatively stable between 1.4–1.9%, the effect of cold increases from 0.7% to 4.7% as the lag period increases. **(**Figure S8). The stratified analysis by 5-year periods showed that the attributable mortality fraction due to non-optimal temperature for the period 2015–2019 was 3.09% (95% CI 1.60 to 4.33) which was slightly lower compared to 3.23% (95% CI 1.59 to 4.62) for the period 2010–2014 (Table S4). Nevertheless, the attributable mortality fraction due to heat for 2015–2019 was 2.68% (95% CI 1.29 to 3.94) which was slightly higher compared to 2.37% (95% CI 1.54 to 3.14) for 2010–2014. When including PM_2.5_ as a potential confounder, we estimate a slight increase in the attributable mortality fractions, compared to our main model, but results remain relatively stable and the relative position of our estimates (e.g., heat vs. cold, 1 st percentile vs. 99th percentile, etc.) remain unchanged (Table S5).

## Discussion

This analysis of the association between ambient temperature and mortality is the first to provide estimates of exposure–response risk curves for non-optimal temperatures and attributable mortality due to heat and cold at a subnational level in Colombia. Our results showed that the exposure–response curves of mortality-temperature association vary by department, and most departments showed acute heat effects but no acute cold effects on daily mortality during the 7-day lag period. The risk of death for heat was higher during the same day exposure and statistically significant for up to 3 days. Moreover, our findings show that the temperature during days warmer than the optimum temperature (heat days) was responsible for 1.77% of acute mortality during 2010–2019 and that the attributable mortality was more than two times higher for heat than for cold temperatures (days colder than the optimum temperature).

Although our main analysis prioritized the assessment of acute effects at a shorter lag period, we found stronger cumulative impacts for cold temperatures at longer lags (up to 4.68% of attributable deaths during the 28-lag period), while those for heat remained relatively stable (1.88% of attributable deaths during the 28-lag period). The differential lag structure observed for heat- and cold-related mortality is consistent with known physiological mechanisms. Heat exposure typically leads to acute health effects—such as heatstroke, dehydration, and cardiovascular stress—that manifest rapidly, often within a few days (Desai et al. 2023). This explains the relatively stable cumulative heat-related mortality risk across different lag periods. In contrast, the health effects of cold exposure tend to emerge more gradually. Cold can exacerbate underlying cardiovascular and respiratory conditions through mechanisms like vasoconstriction, increased blood pressure, and immune suppression, which can lead to infections and complications that develop over time. These delayed physiological responses likely contribute to the progressively higher cold-related mortality observed with longer lag periods (Analitis et al. 2008). These results underscore the need to consider both short and long lag periods when intending to capture the total effect of non-optimal temperatures.

Our results for Colombia, a tropical country on the equator, differ from those reported by the multi-country study that included a two-stage analysis in 304 cities and 13 countries in which it was found that most of this mortality burden was caused by cold days (7.29%), compared with heat days (0.42%) (Gasparrini et al. 2015). The main difference with our results is due to our selection of a short 7-day lag period in our main analysis to account for acute effects of non-optimal temperatures. However, in our sensitivity analysis (see Supplementary Table S3), when using the same lag period used in the multi-country study (21-days lag), we also estimated a higher mortality attributable fraction for cold (3.46%) compared to heat (1.64%). Nevertheless, we estimated a lower magnitude for cold and higher magnitude for heat compared to the pooled estimate for the 13 countries. The differences with our results can also be explained by the fact that almost all the countries included in the study, except Thailand, are located outside the tropical zone where the effect of cold seasons with very low temperature have shown to have a greater effect on mortality. For Thailand, the mortality attributable to cold (2.61%) was higher than that reported for heat (0.76%), the latter being higher than the pooled estimate but lower to that found for our study using the same 21-day lag period.

Our results by department showed that except for Bogotá, Caldas, Cauca, Nariño, and Tolima, the MMP values are below the 50th percentile which is lower than the median MMP reported for most countries (around 80th and 90th percentiles), and even lower that the MMP for the tropical and subtropical areas of Brazil, Taiwan, and Thailand (near the 60th percentile). In contrast to most exposure–response curves for temperature-mortality associations in the multi-country studies that usually exhibit a U-shape (Gasparrini et al. 2015; Zhao et al. 2021), the exposure–response curves for Colombia and its departments tend to show more of a J-shape reflecting the higher risks of acute mortality related to heat compared to cold days. However, this might not be the case when using longer lag periods (21- and 28-days) where the effect of cold temperatures seems to last for up to a few weeks and the number of cold-related attributable deaths is higher than those for heat.

Another multi-country analysis using data from the Global Burden of Disease (GBD) study analyzed the effect of non-optimal temperatures on mortality in 9 countries including Colombia and showed that although in most regions cold effects dominate, locations with high prevailing temperatures can exhibit substantial heat effects exceeding the cold-attributable burden (Burkart et al. 2021). For Colombia, using mortality data from 2001–2005, the estimated attributable mortality fraction due to non-optimal temperature in 2019 was 1.96% which is slightly lower to our findings. A possible explanation of the difference with our findings might be the administrative unit used in that analysis (municipalities) and the fact that increasing temperatures in Colombia during the last decade might have shifted the risk curve to the heat-related mortality. Our results stratified by 5-year periods point towards that direction with a slightly higher total attributable mortality fraction due to heat during the period 2015–2019 (2.68%) compared to 2010–2014 (2.37%). A recent assessment of the impact of non-optimal temperatures was conducted in Colombia at a subnational level during 2010–2019 using estimations of risks for Colombia derived from the GBD study for 17 causes of death (Osorio-García et al. 2023). This report estimated that 1.07% of total deaths for included causes were attributable to non-optimal temperatures, lower than the results obtained in our study. The difference might be explained by the inclusion of all-causes of death and the calculation of attributable fraction based on relative risk estimated from exposure–response curves specific for each department in our study.

One more multi-country study of heat waves and mortality was conducted in 400 communities in 18 countries/regions including Colombia (5 cities with mortality data for 1998–2013) and used a range of heat wave definitions based on the higher percentiles of the communities’ temperature distributions (Guo et al. 2017). The results showed that the relative risk of heat waves on mortality peaked during the same day and lasted up to 3 days and that heat waves had a higher association with mortality in moderate cold and moderate hot areas compared to cold and hot areas. This is consistent with our findings related to the exposure-lag response and heat effects by department. Our results showed that most deaths were caused by exposure to moderately hot temperatures, and the contribution of hotter days was comparatively low. For instance, the attributable fraction of mortality due to days with mean temperature over the 95th percentile was responsible for only 0.29% of deaths. This finding is also in line with results reported for heat and cold temperatures in multi-country studies (Gasparrini et al. 2015; Guo et al. 2017).

There are few studies assessing the association between heat and mortality conducted in Latin America and the Caribbean (LAC) (Bell et al. 2008; Chesini et al. 2022; Méndez-Lázaro et al. 2018; Silveira et al. 2023; Son et al. 2016). A recent study reported the association between non-optimal temperatures and all-cause and cause-specific mortality in 326 cities and 9 countries (which did not include Colombia) between 2002–2015 (Kephart et al. 2022). The study, using a 21-day lag period, estimated that 5.75% of total deaths were attributable to non-optimal temperature, and that the excess death fraction was higher for cold-related days (5.09%) than for heat-related days (0.67%). This attributable fraction for heat is about half of that estimated in this study for Colombia (21-day lag) while for cold temperatures it is about 50% higher. The difference with our findings might be explained by the cold-related deaths in subtropical areas such as south Brazil and Argentina that account for an important number of cities and deaths. Other multi-country studies conducted in LAC (not including Colombia) showed that there is not a clear modifying effect of heat effects by level of green space (Schinasi et al. 2023) or socioeconomic deprivation (Bakhtsiyarava et al. 2023). This coincides with our findings, in which we find that departmental multidimensional poverty does not have a significant effect on mortality risk estimates due to non-optimal temperatures.

Differences in latitude within the same region or even within the same country have shown differential associations between temperature on mortality. For instance, a study conducted in the Brazil Amazonia (Silveira et al. 2023) showed that days with extreme heat were associated with a higher risk of mortality from non-external causes and particularly cardiovascular diseases. On the other hand, a study conducted in Sao Paulo, south Brazil (Son et al. 2016), reported that mortality was more associated with extreme cold days, and in fact the cold effect was higher than the heat effect. This suggests that temperature-mortality associations need to be analyzed at subnational level to account for differences in the risk of death due to non-optimal temperatures.

Our findings showed that even in more homogeneous regions within a tropical country like Colombia, the associations between heat and mortality differ across areas. Moreover, our results by department showed that places with higher mean temperature do not necessarily exhibit the largest effect of heat on mortality. For instance, the departments of Córdoba, Sucre, La Guajira, and Atlántico, which are in the north coast and exhibited the highest MMT did exhibit lower mortality attributable fractions due to heat compared to Meta or Casanare which also showed relatively high mean temperatures but are in the center of the country. This finding may suggest that in tropical areas the effect of heat might be smoothed by the effect of ocean winds or fastest heat-adaptation, a hypothesis that merits further analysis.

Previous multi-country analyses have also estimated that people living in moderate hot areas are more sensitive to heat waves than those living in hot areas which suggest a possible heat stress adaptation (Guo et al. 2017). There are many potential biological mechanisms for adaptation of heat stress including adapted thermoregulation through increased sweat and evaporation that might be modified by intrinsic factors (morphology, heat adaptation, biological sex, and age) (Cramer et al. 2022). Therefore, it is possible that the long-term exposure to high temperatures in specific populations in tropical countries like Colombia might induce an adapted thermoregulation over time that can mitigate the effects of heat. There is evidence that adaptation to heat has been increasing over time and that in highly urbanized populations like cities in the LAC region, the anticipated increase in temperature has implications for future urban planning (Arbuthnott et al. 2016). Our study indicates that, when assessing the acute effect of non-optimal temperature on mortality, more attention should be paid to heat-related associations in moderate hot areas across the country and particularly within urban areas.

In addition to the potential effects related to the lack of ocean winds and potential differences in heat-adaptation, there might be other potential explanations to the higher mortality attributable fractions due to heat in the departments of Meta, Caquetá, and Casanare. The social vulnerability index across departments in Colombia (Durán-Gil 2017) shows that these departments have medium to high vulnerability which can make their population more susceptible to the effects of high temperatures in relation to the proportion of children and older adults, education and health literacy, disperse rural areas, and poverty. However, there are other departments with higher social vulnerability that showed lower mortality attributable fractions due to heat such as the departments of Chocó, Vaupés, and Guainía. A common geographical characteristic of the departments with higher attributable fractions is that all of them are located at the center of the country and east to the mountain region, which suggest that topographic and environmental conditions might be playing an important role in the heat-adaptation to changes in temperature.

One of the key strengths of our study lies in the temporal scope, encompassing a decade of analysis. Additionally, the inclusion of all continental departments in the country enhances the depth and breadth of our findings, providing a detailed characterization of the impact of non-optimal temperatures at the subnational level. Despite these strengths, our study is subject to certain limitations. First, the utilization of temperature data derived from ERA5 introduces a potential source of measurement error when compared to surface temperature. While ERA5 is widely acknowledged as the standard source for satellite-based temperature estimations at the surface level and is commonly employed in large-scale temperature effect studies, it is crucial to acknowledge the inherent limitations associated with remote sensing. Second, the aggregation of data to the departmental level may present limitations in capturing the differential effects of non-optimal temperatures in smaller geographical areas. The use of departments as a broad administrative unit might lead to the dilution of the effects of cold or heat-related mortality in specific municipalities characterized by colder or hotter temperature conditions. Third, we used the MPI for 2018 as the poverty index of reference for the entire study period which might introduce some uncertainty. The MPI for Colombia decreased from 29.7 in 2010 to 17.5 in 2019 and despite the exact quantitative index for each department might have changed over the decade, the geographical distribution of poverty remains similar across regions and departments. To date, the MPI 2018 is the most reliable index as it was built based on census data, which reduces the uncertainty of this socioeconomic measure (Departamento Nacional de Estadística (DANE) 2022).

Also, we acknowledge the existing limitations in obtaining good air pollution exposure data across the county and therefore relying on modelled PM_2.5_ from Copernicus which also has a coarse spatial resolution. Our main models did not control for PM_2.5_ as a potential confounder of temperature effects as we agree with causal models that locate air pollution as a mediator in the causal pathway (Buckley et al. 2014). Nevertheless, as there is still debate about the need for controlling for air pollution in temperature studies (Hu et al. 2022), we conducted a sensitivity analysis controlling for PM_2.5_. Our results showed that the inclusion of PM_2.5_ as a potential confounder does not change the interpretation of our results.

The implications of our research extend far beyond the immediate findings, offering valuable insights that can inform targeted adaptation strategies at a regional level. The identification of department-specific variations in the impact of non-optimal temperatures underscores the necessity for adaptation measures that are tailored to the unique vulnerabilities of each region. Our study highlights the importance of prioritizing acute heat-related effects in areas characterized by moderate temperatures across the country but also emphasizes the need to consider longer lag periods when intending to capture the total effect of hot and cold non-optimal temperatures. This information is crucial for public health planning and resource allocation, as it emphasizes the need for focused interventions to mitigate the potential substantial impact on the well-being of local populations. Moving forward, further investigations at smaller geographical scales, such as municipalities and intraurban variations within major cities, are imperative. Analyzing temperature effects at these finer resolutions will allow for a better understanding of the localized dynamics, enabling the development of targeted interventions and policies. Additionally, exploring the impact of non-optimal temperatures on specific age groups and causes of mortality and morbidity will provide a more comprehensive perspective on less severe effects, facilitating the implementation of preventive measures that address the unique vulnerabilities of different demographic groups.

## Conclusions

Understanding the characteristics and variation of temperature-mortality association at a national and subnational level is important for informing and protecting public health facing the challenges of temperature rises and climate change. To our knowledge this is the first study to assess the association of non-optimal temperature on all-cause mortality and to estimate exposure–response curves at a subnational level in Colombia. Our findings suggest that non-optimal temperatures vary across departments and are responsible for a relatively low fraction of the total mortality. The non-optimal acute attributable mortality (0–7 days) is mainly related to heat while the subacute attributable mortality (14 to 28 days) is mainly related to cold. The mortality risk associated with heat is higher during the same day and the effects lasted predominantly for 3 days and seem to be stronger in populations living in places with moderate hot temperatures. The observed variation of heat-related mortality across departments indicates the importance of developing local adaptation and response plans.

## Supplementary Material

Appendix **Supplementary Information** The online version contains supplementary material available at https://doi.org/10.1007/s11869-025-01782-9.

## Figures and Tables

**Table 1:** Attributable deaths and mortality fraction due to acute non-optimal temperatures in Colombia, 2010–2019

Non-optimal temperature categories	Attributable deaths N (95% CI)	Attributable fraction % (95% CI)
Total*	62,822 (36,269 to 84,536)	2.45 (1.42 to 3.30)
Cold*	17,382 (−3,796 to 38,187)	0.68 (−0.15 to 1.49)
Heat*	45,440 (29,618 to 59,198)	1.77 (1.16 to 2.31)
1 st percentile	773 (−161 to 1,431)	0.03 (−0.01 to 0.06)
5th percentile	2,648 (707 to 4,414)	0.10 (0.03 to 0.17)
10th percentile	4,499 (795 to 7,362)	0.18 (0.03 to 0.29)
90th percentile	12,882 (9,981 to 15,735)	0.50 (0.39 to 0.61)
95th percentile	7,451 (5,964 to 8,789)	0.29 (0.23 to 0.34)
99th percentile	2,053 (1,615 to 2,490)	0.08 (0.06 to 0.10)

* Calculated based on best linear unbiased predictor as the optimal temperature value for each department. Cold and Heat attributable mortality were calculated for days with mean temperature below and above the optimal temperature, respectively, for the main model using 7-days lag

**Table 2:** Attributable deaths and attributable mortality fraction due to non-optimal temperatures by departments in Colombia, 2010–2019

Department	MMT	MMP	Attributable deaths (N- 95%CI)				Attributable fraction (%—95% CI)		
			Overall	Cold	Heat		Overall	Cold	Heat
Amazonas	22.96	1	82 (−105 to 257)	−1 (−9 to 3)	83 (−137 to 266)		3.58 (−4.59 to 11.25)	−0.05 (−0.38 to 0.13)	3.63 (−6.01 to 11.68)
Antioquia	22.54	23	3,860 (−694 to 8,217)	241 (−832 to 1,281)	3,619 (−1,299 to 8,181)		1.06 (−0.19 to 2.26)	0.07 (−0.23 to 0.35)	1.00 (−0.36 to 2.25)
Arauca	22.84	1	433 (−748 to 1,500)	0 (−11 to 8)	434 (−749 to 1,593)		2.59 (−4.48 to 8.97)	0.00 (−0.06 to 0.05)	2.59 (−4.48 to 9.53)
Atlántico	26.22	2	2,188 (−2,966 to 6,819)	5 (−84 to 85)	2,184 (−2,698 to 7,118)		1.87 (−2.54 to 5.84)	0.00 (−0.07 to 0.07)	1.87 (−2.31 to 6.10)
Bogotá, DC	14.29	99	10,491 (−8,725 to 29,176)	10,479 (−8,700 to 31,113)	12 (−187 to 173)		2.65 (−2.21 to 7.38)	2.65 (−2.20 to 7.87)	0.00 (−0.05 to 0.04)
Bolívar	25.51	7	2,064 (−494 to 4,588)	30 (−129 to 183)	2,033 (−767 to 4,684)		2.43 (−0.58 to 5.40)	0.04 (−0.15 to 0.22)	2.39 (−0.90 to 5.51)
Boyacá	15.52	34	711 (−193 to 1,620)	190 (−301 to 639)	522 (−311 to 1,363)		1.03 (−0.28 to 2.34)	0.27 (−0.44 to 0.92)	0.75 (−0.45 to 1.97)
Caldas	20.18	64	661 (79 to 1,227)	397 (−247 to 999)	264 (61 to 485)		1.05 (0.13 to 1.94)	0.63 (−0.39 to 1.58)	0.42 (0.10 to 0.77)
Caquetá	21.98	1	1,110 (−555 to 2,596)	−6 (−28 to 11)	1,116 (−575 to 2,637)		5.56 (−2.78 to 13.01)	−0.03 (−0.14 to 0.06)	5.59 (−2.88 to 13.22)
Casanare	23.27	1	704 (−156 to 1,576)	−6 (−20 to 3)	711 (−241 to 1,589)		4.91 (−1.09 to 11.00)	−0.04 (−0.14 to 0.02)	4.96 (−1.68 to 11.09)
Cauca	20.29	95	904 (−1,769 to 3,282)	826 (−1,661 to 3,069)	79 (−18 to 178)		1.40 (−2.74 to 5.08)	1.28 (−2.57 to 4.75)	0.12 (−0.03 to 0.28)
Cesar	23.20	1	1,900 (−1,691 to 5,290)	−11 (−36 to 13)	1,911 (−1,762 to 5,236)		4.32 (−3.84 to 12.02)	−0.02 (−0.08 to 0.03)	4.34 (−4.01 to 11.90)
Chocó	25.12	25	258 (2 to 480)	42 (−40 to 118)	216 (−36 to 468)		1.74 (0.01 to 3.24)	0.28 (−0.27 to 0.80)	1.46 (−0.24 to 3.15)
Córdoba	26.61	23	999 (−45 to 2,011)	82 (−180 to 302)	917 (−171 to 2,098)		1.11 (−0.05 to 2.23)	0.09 (−0.20 to 0.34)	1.02 (−0.19 to 2.33)
Cundinamarca	18.44	48	1,152 (−155 to 2,395)	522 (−591 to 1,471)	630 (−416 to 1,633)		0.78 (−0.10 to 1.61)	0.35 (−0.40 to 0.99)	0.42 (−0.28 to 1.10)
Guainía	24.71	26	41 (−3 to 77)	4 (−9 to 16)	37 (−2 to 77)		2.69 (−0.17 to 5.04)	0.26 (−0.59 to 1.05)	2.43 (−0.11 to 5.04)
Guaviare	22.36	1	174 (−176 to 469)	0 (−3 to 2)	175 (−154 to 489)		4.42 (−4.45 to 11.89)	−0.01 (−0.08 to 0.06)	4.43 (−3.89 to 12.39)
Huila	17.92	4	1,255 (−1,656 to 4,303)	9 (−166 to 159)	1,246 (−1,874 to 4,156)		1.63 (−2.14 to 5.57)	0.01 (−0.22 to 0.21)	1.61 (−2.43 to 5.38)
La Guajira	26.23	40	383 (−8 to 736)	125 (−114 to 337)	258 (−92 to 594)		1.67 (−0.03 to 3.21)	0.55 (−0.50 to 1.47)	1.12 (−0.40 to 2.59)
Magdalena	25.52	23	751 (−38 to 1,520)	23 (−180 to 226)	728 (−209 to 1,633)		1.29 (−0.06 to 2.61)	0.04 (−0.31 to 0.39)	1.25 (−0.36 to 2.80)
Meta	22.12	1	3,599 (−56 to 6,840)	−11 (−51 to 24)	3,610 (−109 to 7,138)		5.66 (−0.09 to 10.75)	−0.02 (−0.08 to 0.04)	5.67 (−0.17 to 11.22)
Nariño	22.16	98	2,579 (−2,562 to 7,052)	2,566 (−1,782 to 6,995)	12 (−47 to 71)		3.02 (−3.00 to 8.27)	3.01 (−2.09 to 8.20)	0.01 (−0.06 to 0.08)
Norte de Santander	20.28	7	2,256 (−356 to 4,691)	110 (−36 to 255)	2,145 (−196 to 4,503)		2.82 (−0.45 to 5.87)	0.14 (−0.05 to 0.32)	2.69 (−0.25 to 5.64)
Putumayo	21.44	1	825 (−529 to 1,910)	−5 (−19 to 6)	830 (−341 to 2,004)		5.28 (−3.38 to 12.23)	−0.03 (−0.12 to 0.04)	5.31 (−2.18 to 12.83)
Quindío	15.53	7	1,549 (−20 to 3,139)	72 (−36 to 179)	1,477 (−64 to 2,963)		3.81 (−0.05 to 7.73)	0.18 (−0.09 to 0.44)	3.63 (−0.16 to 7.29)
Risaralda	17.96	5	2,124 (−173 to 4,519)	47 (−102 to 185)	2,078 (−458 to 4,164)		3.30 (−0.27 to 7.02)	0.07 (−0.16 to 0.29)	3.23 (−0.71 to 6.47)
Santander	20.57	6	4,070 (445 to 7,431)	102 (−93 to 284)	3,968 (830 to 7,490)		3.60 (0.39 to 6.58)	0.09 (−0.08 to 0.25)	3.51 (0.73 to 6.63)
Sucre	27.84	43	565 (125 to 1,014)	129 (−196 to 439)	436 (38 to 778)		1.36 (0.30 to 2.43)	0.31 (−0.47 to 1.05)	1.05 (0.09 to 1.87)
Tolima	21.30	76	1,595 (−435 to 3,482)	969 (−832 to 2,923)	625 (142 to 1,088)		1.72 (−0.47 to 3.76)	1.05 (−0.90 to 3.15)	0.67 (0.15 to 1.17)
Valle del Cauca	19.97	6	13,436 (4,007 to 22,784)	431 (−75 to 914)	13,005 (3,377 to 21,583)		4.64 (1.38 to 7.87)	0.15 (−0.03 to 0.32)	4.49 (1.17 to 7.45)
Vaupés	24.37	27	38 (−3 to 74)	5 (−7 to 16)	33 (−6 to 67)		3.05 (−0.21 to 6.00)	0.41 (−0.54 to 1.29)	2.64 (−0.50 to 5.47)
Vichada	25.69	36	64 (10 to 116)	15 (−15 to 43)	49 (−1 to 98)		2.52 (0.39 to 4.56)	0.58 (−0.57 to 1.69)	1.93 (−0.02 to 3.82)
**Colombia**	18.9	15	**62,822** **(36,269 to** **84,536)**	**17,382** **(−3,796 to** **38,187)**	**45,440** **(29,618 to** **59,198)**		**2.45** **(1.42 to 3.30)**	**0.68** **(−0.15 to 1.49)**	**1.77** **(1.16 to 2.31)**

*MMT* Minimum Mortality Temperature; *MMP* Minimum Mortality Percentile

## Data Availability

The datasets generated during and/or analyzed during the current study are available from the corresponding author on reasonable request.
