# Supplementary material for: The effect of non-optimal ambient temperature on daily mortality in Colombia 2010–2019
Source: Air Qual Atmos Health. Author manuscript; Available in PMC 2025 Sep 13. (PMC7618110; doi:10.1007/s11869-025-01782-9)
Supplement: Appendix — Supplementary Information The online version contains supplementary material available at https://doi.org/10.1007/s11869-025-01782-9. [file EMS208664-supplement-Appendix.pdf]

Supplementary Material

The effect of non-optimal ambient temperature on daily mortality in Colombia 2010-2019

Nicolas Borchers-Arriagada[1], Antonio Gasparrini[2] Laura A. Rodriguez-Villamizar[3]

1. Menzies Institute for Medical Research, University of Tasmania, Tasmania, Australia
2. Environment & Health Modelling (EHM) Lab, Department of Public Health Environments and Society, London School of Hygiene & Tropical Medicine, London, UK
3. Departamento de Salud Pública, Escuela de Medicina, Universidad Industrial de Santander, Carrera 32 29-31 Of. 301, 680002, Bucaramanga, Colombia

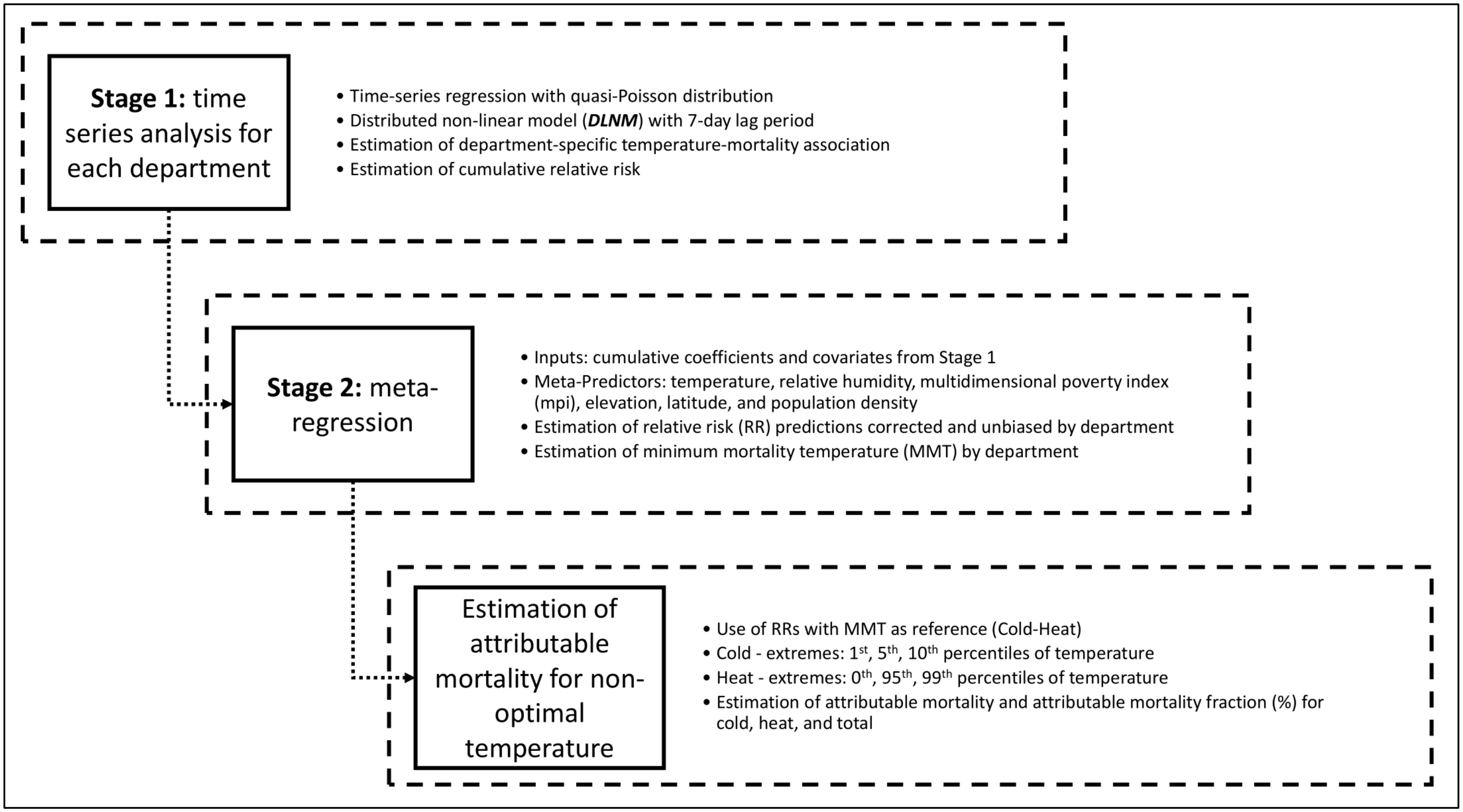

Figure S1. Summarised methods

Table S1. Descriptive statistics by departments of Colombia, 2010-2019 (3,652 days)

Department	Deaths (*)(**) (#)	Mean population (#)	Mean annual mortality rate (#deaths per 100.000)	Temperature (**) (°C)	Relative humidity (**) (%)	$PM_{2.5}$ (**) (µg/m^3)	Multidimensional poverty index (***)	Population-weighted Latitude (degrees)	Population-weighted Elevation (m)
Antioquia	99.4 (12.5)	6,127,335	5,923	23.3 (0.9)	81.8 (5.4)	16.7 (9.2)	15.3	6.5	1,456
Atlántico	32 (8.4)	2,403,672	4,856	28.1 (0.9)	80.6 (6.2)	23.3 (13)	21.1	10.9	42
Bogota, D.C.	108.3 (11.8)	7,284,377	5,430	12.4 (0.7)	78.5 (8.4)	25 (7.2)	4.1	4.6	2,626
Bolivar	23.3 (6.3)	1,996,056	4,255	27.2 (1.2)	82.2 (7.1)	20.6 (14.6)	31.9	9.8	64
Boyacá	18.9 (4.4)	1,192,985	5,796	15.8 (0.7)	80.4 (5.4)	15.6 (6.3)	16.5	5.7	2,321
Caldas	17.3 (4.3)	988,572	6,392	19.9 (0.9)	74.4 (9.2)	22.1 (6.1)	13.8	5.2	1,649
Caquetá	5.5 (2.5)	397,876	5,013	24.5 (1.1)	86.7 (6.2)	14.8 (16.3)	26.9	1.5	510
Cauca	17.7 (4.9)	1,414,656	4,570	19.2 (0.6)	85.3 (3.9)	13.1 (4.6)	27.7	2.6	1,649
Cesar	12 (3.9)	1,117,111	3,938	26 (1.2)	75.5 (9.5)	21.3 (14.4)	31.7	9.7	282
Cordoba	24.6 (6)	1,719,439	5,234	27.4 (1)	82.6 (5.8)	16.8 (9.1)	34.4	8.7	69
Cundinamarca	40.6 (7.1)	2,538,931	5,844	18.5 (0.7)	76.8 (6.6)	23 (6.5)	10.5	4.7	2,240
Choco	4.1 (2.1)	502,932	2,952	25.7 (0.8)	87.9 (3.5)	12.3 (5.8)	46.3	5.8	295
Huila	21.1 (5)	1,056,081	7,311	19.3 (0.8)	75.9 (7.2)	15.8 (8.7)	18.0	2.5	1,104
La Guajira	6.3 (3)	805,324	2,847	26.6 (1.1)	77.3 (5.1)	18.6 (10.7)	53.3	11.4	118
Magdalena	16 (4.8)	1,270,134	4,592	26.5 (1.2)	78.3 (8.9)	21.9 (14.8)	36.4	10.6	118
Meta	17.4 (4.5)	977,280	6,511	24.7 (1.3)	80.6 (10)	17.9 (11.9)	15.6	3.8	447
Nariño	23.3 (5.3)	1,603,677	5,317	20.9 (0.6)	86.7 (3.1)	12.1 (4.1)	33.1	1.4	1,865
Norte de Santander	21.9 (6.4)	1,425,392	5,605	21.7 (1)	77.6 (6.5)	21.1 (10.6)	29.5	8.0	724
Quindío	11.1 (3.4)	528,134	7,693	16.6 (0.8)	83.7 (7.1)	22.9 (6.8)	14.2	4.5	1,441
Risaralda	17.6 (4.4)	924,090	6,967	19.1 (0.7)	88 (3.3)	19.6 (5.4)	11.6	4.9	1,430
Santander	30.9 (6.5)	2,097,330	5,385	21.9 (0.9)	81.2 (5.7)	19.9 (8.9)	12.6	6.9	994
Sucre	11.4 (3.8)	868,212	4,798	28.2 (1.2)	81.6 (7)	19.8 (11.3)	41.7	9.2	114
Tolima	25.4 (5.4)	1,321,579	7,011	20.6 (1)	68.4 (10.1)	21.7 (6.7)	22.2	4.3	1,049
Valle del Cauca	79.3 (11.9)	4,400,738	6,580	21 (0.7)	86.6 (2.8)	16.4 (5.4)	14.1	3.6	994
Arauca	4.6 (2.4)	243,937	6,853	25.4 (1.3)	78.4 (11.4)	18.3 (10.6)	27.6	6.8	304
Casanare	3.9 (2.1)	393,903	3,638	26.1 (1.4)	76.7 (12)	17 (8.9)	19.0	5.3	410
Putumayo	4.3 (4.2)	325,403	4,800	24.1 (1.2)	87.3 (5.1)	14.4 (12.4)	24.1	0.7	629
Amazonas	0.6 (0.8)	71,892	3,171	25.3 (1)	89 (3.7)	10.8 (4.6)	35.4	-3.1	115
Guainía	0.4 (0.7)	42,865	3,581	25.3 (1)	89 (4.7)	12.2 (6)	60.6	3.2	129
Guaviare	1.1 (1.1)	77,643	5,085	24.9 (1.1)	87.8 (7)	13.3 (10.2)	31.4	2.3	216
Vaupes	0.3 (0.6)	37,034	3,327	24.9 (0.9)	89.3 (4.2)	11.1 (5.1)	68.5	0.8	200
Vichada	0.7 (0.9)	99,556	2,564	26.2 (1.2)	83.1 (9.6)	15.3 (7.8)	63.5	5.0	113

* All-cause deaths. ** Daily mean (SD) for each Department. ** Multidimensional poverty index based on census data 2018.

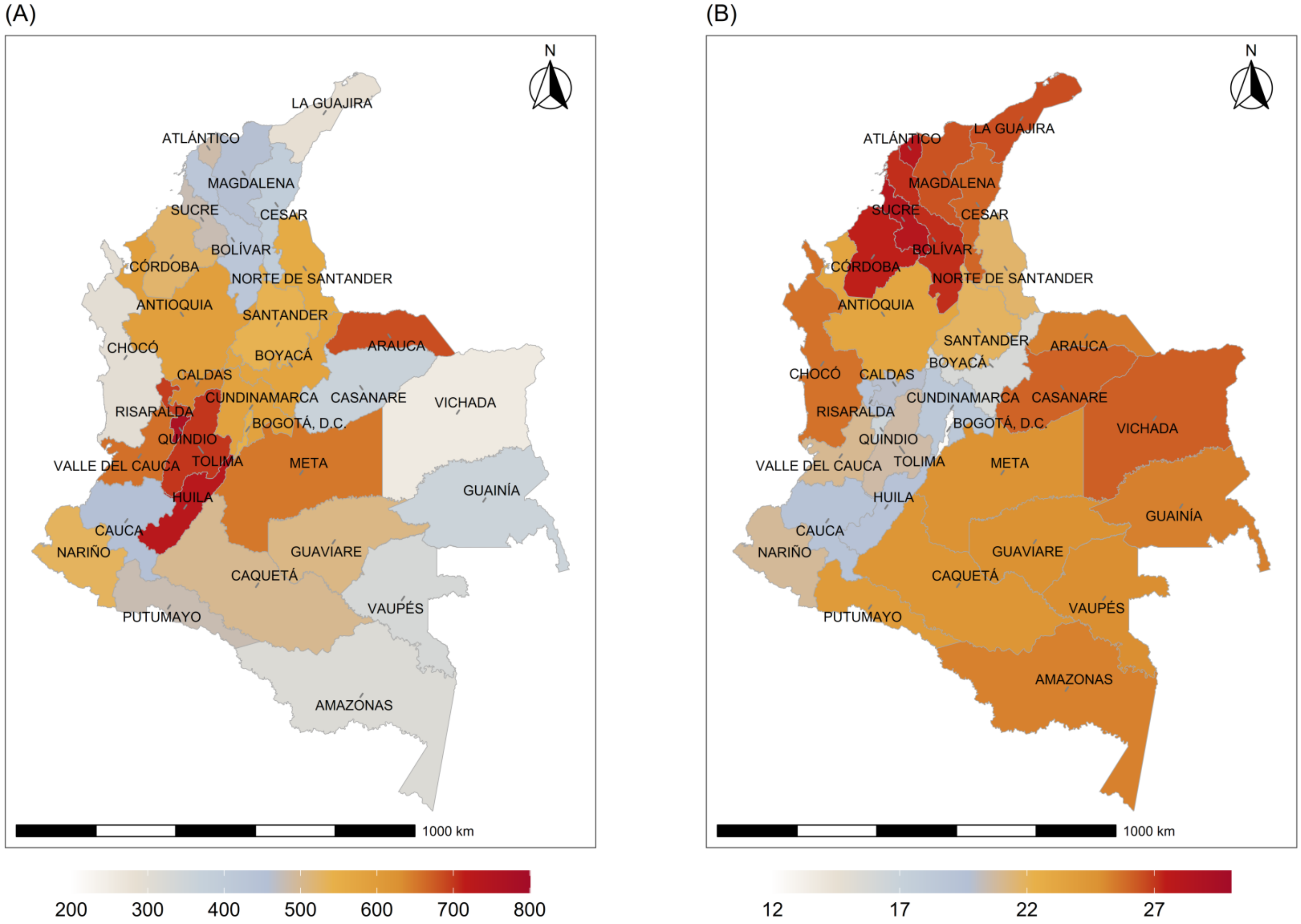

Figure S2. Mean annual statistics by department in Colombia, 2010-2019: (A) mean mortality rate (# deaths per 100,000 persons), and (B) mean ambient temperature (°C)

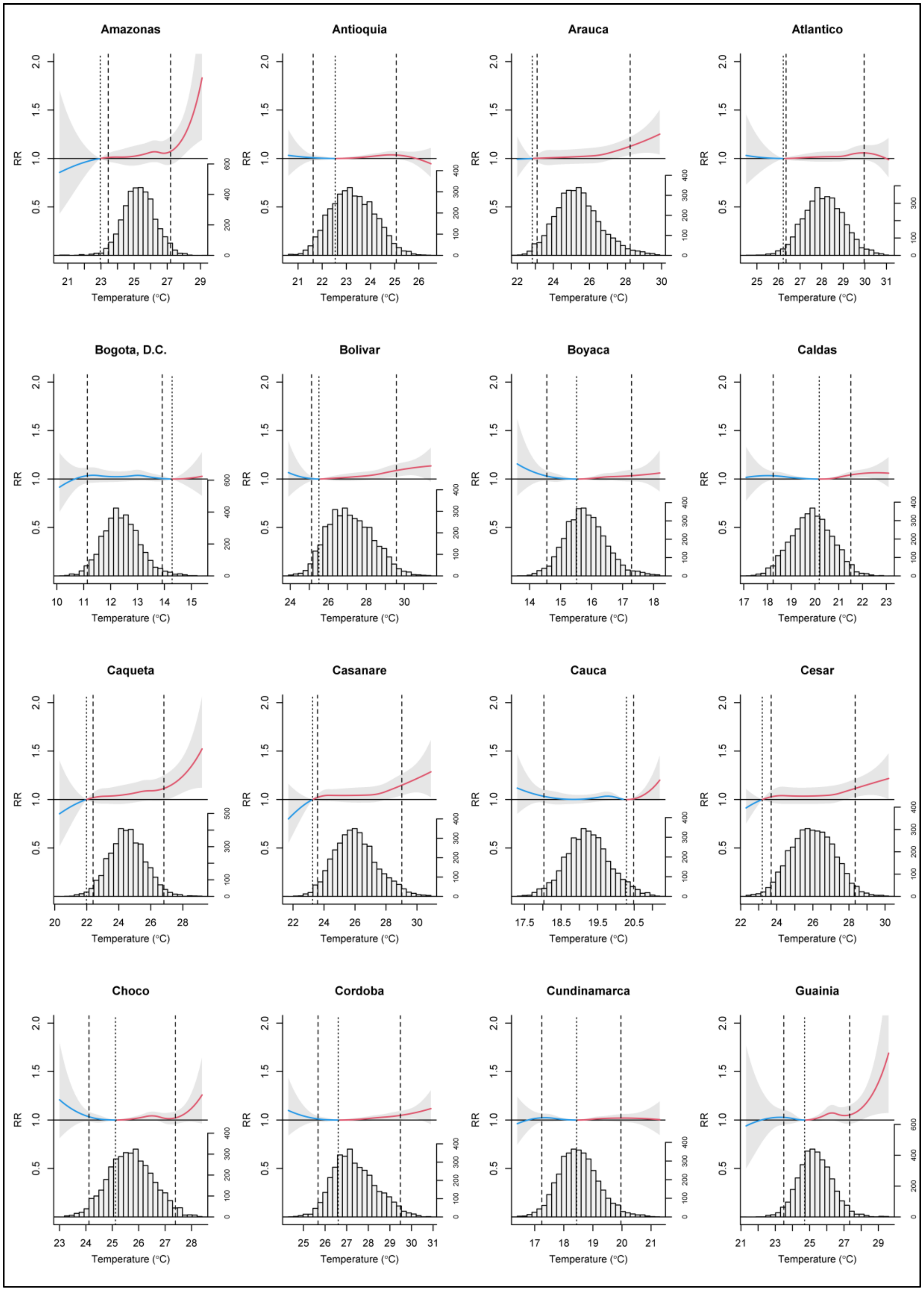

Figure S3. Estimates of overall cumulative exposure-response for selected departments in Colombia, 2010-2019

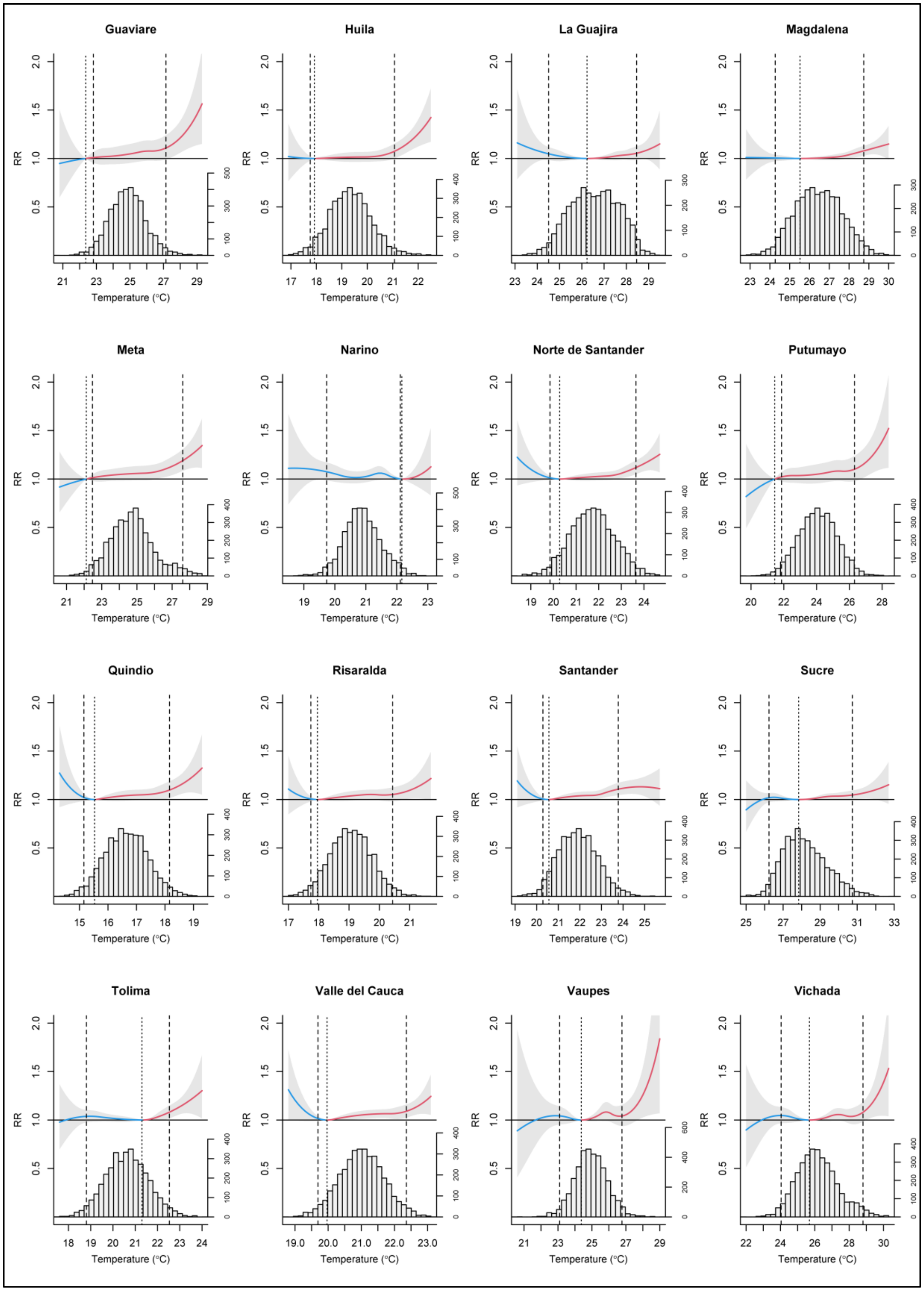

Figure S4. Estimates of overall cumulative exposure-response for selected departments in Colombia, 2010-2019

For Figures S3 and S4 the curves represent the exposure–response associations as best linear unbiased prediction (with 95% empirical CI, shaded grey) with related temperature distributions. Dashed lines are minimum mortality temperatures and dashed black lines are the 2.5th and 97.5th percentiles. RR=relative risk.

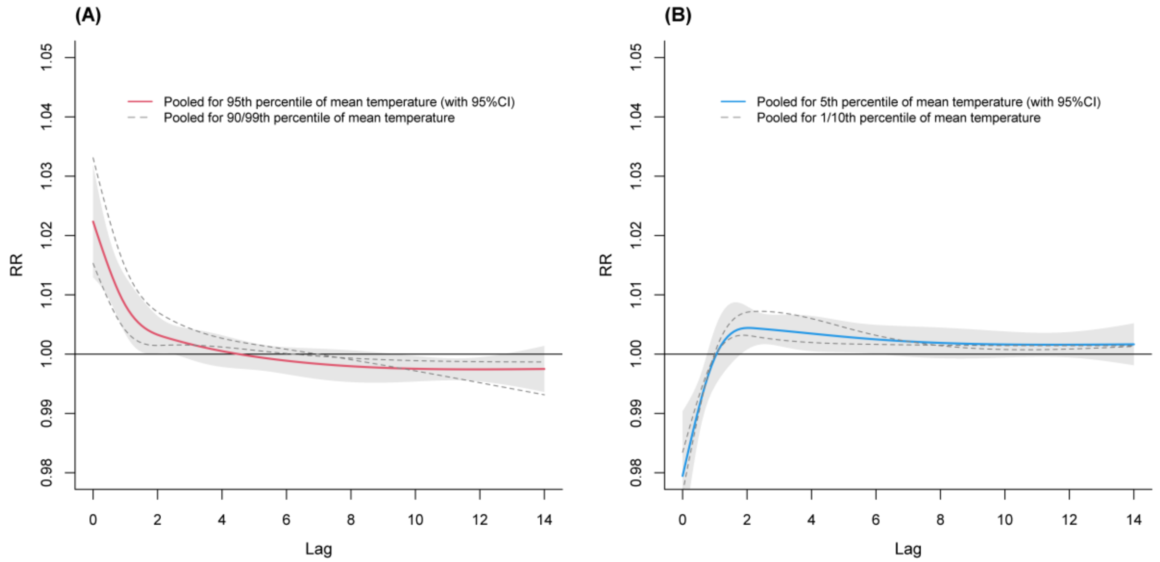

Figure S5. Pooled predictor-specific temperature-mortality association for Colombia using 14-day lag: A) Heat B) Cold.

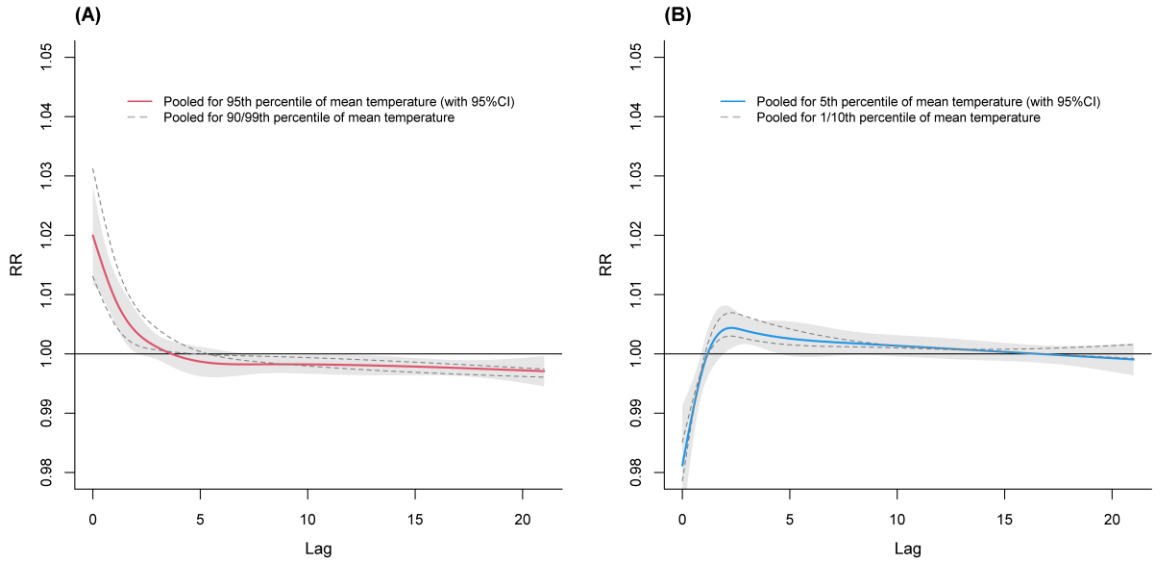

Figure S6. Pooled predictor-specific temperature-mortality association for Colombia using 21-day lag: A) Heat B) Cold.

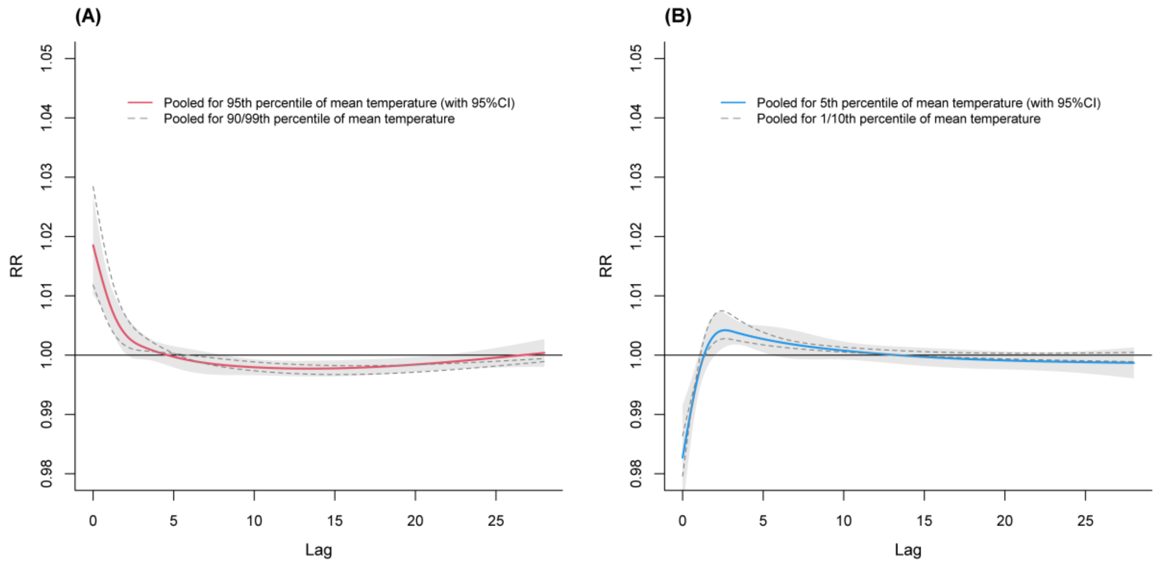

Figure S7. Pooled predictor-specific temperature-mortality association for Colombia using 28-day lag: A) Heat B) Cold.

Table S2. Results of multivariate meta-regression model for temperature-mortality cumulative risk associations by department in Colombia 2010-2019

Meta-Predictors *	Wald Test	Q test	I^2
Average temperature	0.200	124.3, p-value = 0.376	3.4%
Range of temperature	0.582		
Multidimensional poverty index	0.353		
Population weighted latitude	0.756		
Population weighted elevation	0.012		
Average population density	0.359		
Average relative humidity	0.295		

***Results for meta-predictors using main model with 7-day lag and 6 df for seasonality.**

Table S3. Results of sensitivity analyses using different model choices. NOTE: results for main analysis shaded in grey.

Model choice	Attributable deaths (N- 95%CI)			Attributable fraction (% - 95% CI)		
	Overall	Cold	Heat	Overall	Cold	Heat
Lag = 7, dfseas = 4	69,474 (33,694 to 101,547)	19,972 (-7,756 to 44,562)	49,502 (28,898 to 69,324)	2.71 (1.32 to 3.96)	0.78 (-0.30 to 1.74)	1.93 (1.13 to 2.71)
**Main model**	62,822 (36,269 to 84,536)	17,382 (-3,796 to 38,187)	45,440 (29,618 to 59,198)	2.45 (1.42 to 3.30)	0.68 (-0.15 to 1.49)	1.77 (1.16 to 2.31)
Lag = 7, dfseas = 8	68,578 (37,717 to 95,886)	16,463 (-8,933 to 41,457)	52,116 (38,585 to 65,810)	2.68 (1.47 to 3.74)	0.64 (-0.35 to 1.62)	2.03 (1.51 to 2.57)
Lag = 14, dfseas = 4	92,830 (51,316 to 130,381)	52,097 (19,991 to 81,490)	40,733 (19,296 to 61,011)	3.62 (2.00 to 5.09)	2.03 (0.78 to 3.18)	1.59 (0.75 to 2.38)
Lag = 14, dfseas = 6	87,960 (50,759 to 122,774)	51,524 (18,835 to 79,355)	36,436 (15,613 to 53,950)	3.43 (1.98 to 4.79)	2.01 (0.74 to 3.10)	1.42 (0.61 to 2.11)
Lag = 14, dfseas = 8	96,162 (46,518 to 140,956)	45,293 (2,863 to 83,014)	50,869 (21,989 to 74,544)	3.75 (1.82 to 5.50)	1.77 (0.11 to 3.24)	1.99 (0.86 to 2.91)
Lag = 21, dfseas = 4	127,802 (76,044 to 169,576)	89,534 (47,033 to 122,749)	38,268 (13,567 to 61,417)	4.99 (2.97 to 6.62)	3.50 (1.84 to 4.79)	1.49 (0.53 to 2.40)
Lag = 21, dfseas = 6	130,643 (78,134 to 172,562)	88,705 (50,262 to 121,679)	41,937 (9,864 to 66,788)	5.10 (3.05 to 6.74)	3.46 (1.96 to 4.75)	1.64 (0.39 to 2.61)
Lag = 21, dfseas = 8	133,383 (70,389 to 189,148)	84,062 (27,405 to 131,838)	49,321 (22,672 to 71,816)	5.21 (2.75 to 7.38)	3.28 (1.07 to 5.15)	1.93 (0.89 to 2.80)
Lag = 28, dfseas = 4	158,951 (90,072 to 214,426)	119,432 (64,680 to 169,385)	39,520 (7,196 to 66,274)	6.21 (3.52 to 8.37)	4.66 (2.53 to 6.61)	1.54 (0.28 to 2.59)
Lag = 28, dfseas = 6	168,051 (90,418 to 229,110)	119,808 (59,883 to 172,183)	48,242 (7,625 to 81,413)	6.56 (3.53 to 8.94)	4.68 (2.34 to 6.72)	1.88 (0.30 to 3.18)
Lag = 28, dfseas = 8	170,851 (85,229 to 243,172)	95,062 (8,802 to 160,267)	75,789 (33,367 to 110,366)	6.67 (3.33 to 9.49)	3.71 (0.34 to 6.26)	2.96 (1.30 to 4.31)

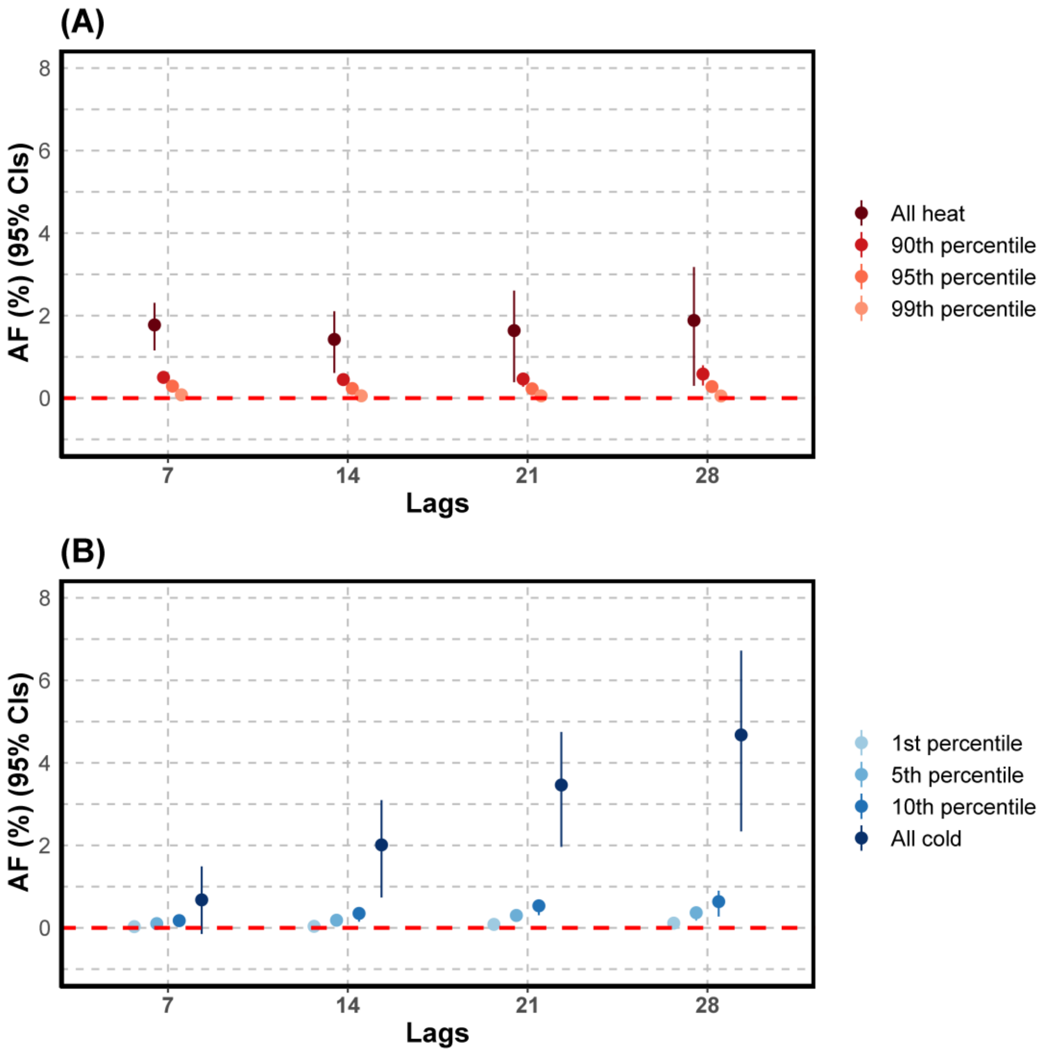

Figure S8. Attributable mortality fraction (%) due to non-optimal temperatures by Lags (df = 6) in Colombia, 2010-2019

Table S4. Attributable mortality fraction due to acute non-optimal temperatures in Colombia: 2010-2019, 2010-2014, and 2015-2019

**Non-optimal temperature categories**	**2010-2019**	**2010-2014**	**2015-2019**
Total*	2.45 (1.42 to 3.30)	3.23 (1.59 to 4.62)	3.09 (1.60 to 4.33)
Cold*	0.68 (-0.15 to 1.49)	0.86 (-0.42 to 2.01)	0.41 (0.08 to 0.71)
Heat*	1.77 (1.16 to 2.31)	2.37 (1.54 to 3.14)	2.68 (1.29 to 3.94)
1st percentile	0.03 (-0.01 to 0.06)	0.06 (0.01 to 0.09)	0.03 (0.00 to 0.05)
5th percentile	0.10 (0.03 to 0.17)	0.18 (0.09 to 0.26)	0.12 (0.07 to 0.16)
10th percentile	0.18 (0.03 to 0.29)	0.29 (0.10 to 0.44)	0.23 (0.13 to 0.32)
90th percentile	0.50 (0.39 to 0.61)	0.52 (0.35 to 0.66)	0.62 (0.45 to 0.78)
95th percentile	0.29 (0.23 to 0.34)	0.28 (0.19 to 0.36)	0.35 (0.25 to 0.44)
99th percentile	0.08 (0.06 to 0.10)	0.07 (0.04 to 0.10)	0.08 (0.05 to 0.11)

Table S5. Attributable mortality fraction due to acute non-optimal temperatures in Colombia (2010-2019, 2010-2014, and 2015-2019): controlling for $PM_{2.5}$ as a potential confounder

Non-optimal temperature categories	2010-2019	2010-2014	2015-2019
Total*	2.59 (1.48 to 3.61)	3.52 (2.38 to 4.59)	3.14 (1.69 to 4.50)
Cold*	0.71 (-0.33 to 1.64)	1.14 (0.11 to 2.00)	0.44 (0.09 to 0.77)
Heat*	1.88 (1.29 to 2.44)	2.38 (1.72 to 2.99)	2.70 (1.35 to 3.99)
1st percentile	0.03 (-0.01 to 0.06)	0.06 (0.02 to 0.09)	0.03 (0.00 to 0.05)
5th percentile	0.11 (0.01 to 0.19)	0.19 (0.11 to 0.27)	0.13 (0.07 to 0.18)
10th percentile	0.19 (0.01 to 0.33)	0.31 (0.19 to 0.44)	0.24 (0.13 to 0.34)
90th percentile	0.51 (0.39 to 0.63)	0.56 (0.45 to 0.66)	0.61 (0.43 to 0.78)
95th percentile	0.29 (0.23 to 0.35)	0.30 (0.23 to 0.36)	0.34 (0.24 to 0.44)
99th percentile	0.08 (0.06 to 0.10)	0.08 (0.05 to 0.10)	0.08 (0.04 to 0.12)
